# Supplementary material for: Global opportunities for mariculture development to promote human nutrition
Source: PeerJ. 2018 May 9;6:e4733. doi: 10.7717/peerj.4733 (PMC5949058; doi:10.7717/peerj.4733)
Supplement: Supplemental Information 1 [file peerj-06-4733-s001.docx]

**Global opportunities for mariculture development to promote human nutrition**

Owen R. Liu, Renato Molina, Margaret Wilson, Benjamin S. Halpern

**APPENDIX**

As part of our global analysis of mariculture opportunity, we performed several extensions and sensitivity analyses to test the robustness of our results. The findings of these additional analyses are introduced in the main text, but are more fully explored here, organized into three main sections: cross-sectional limitations, seafood reliance vs. preference, and sensitivity analyses.

***Cross-sectional limitations***

One of the main limitations in our analysis is the fact that we are not able to deduce trends that contributed to a country’s relative opportunity scores. This issue could be problematic because we could be measuring an effect that is a temporal anomaly or overlooking a trend suggesting that our assessment is expected to change significantly in the short term. Because of the same data limitations which limited our main analysis to cross-sectional data in the first place, we are only able to address this issue for the economic opportunity metrics. Nonetheless, if comprehensive panel data were to become available, a similar extension for nutritional metrics could follow a similar methodology.

To evaluate how sensitive our results are to changes over time, we conducted the same analysis as in the main text, but for several different years. We then evaluated if and how countries changed in terms of their rankings between years. Significant changes in rankings would mean that countries are significantly “reshuffling” between years, or going through essential changes that our analysis is unable to capture by examining just a single time period. If this were true, the validity of our main analysis would be suspect.

Figure A1 shows an example of these comparisons for year 2000 and 2011, with each panel reporting how countries have changed in their relative rankings for all of the economic metrics and the total economic opportunity score. If a point lies on the green line, it means there was no change in that nation’s relative ranking between 2000 and 2011. Points above the green line imply that countries move up in their ranking, while points below indicate drops. From the figure, we see that while there is movement in the ranking, many countries have a relatively stable position across both time periods. We can examine if both rankings are statistically different from each other by performing a rank concordance test (Kendall’s W test; Kendall 1955).

The results of the concordance analysis are reported in Table A1. In particular, we compared metrics and scores for years 2000, 2005, and 2011. The table reports the Kendall’s W and the associated test for statistical significance. The closer the Kendall’s W statistic is to one, the more concordance or agreement there is among the two rankings. According to this analysis, all comparisons across periods and metrics show statistically significant concordance. In other words, across metrics and time periods, countries’ relative positions have remained consistent over the time.

These results give further confidence to the robustness of our main, cross-sectional analysis using only 2011 data. At least for the time periods evaluated, the components of our global economic opportunity metric have remained statistically consistent. This property implies that our main results for 2011 are not a result of sporadic measurements, but rather a state of the world that has had at least ten years of relative consistency.

It would be ideal to expand this analysis and insight to the nutritional metrics. Unfortunately, data limitations—the same that prevented our main analysis from using a panel approach in the first place—do not allow us to perform such analysis and extrapolating these results would be inappropriate. This approach, however, is straightforward. If a richer dataset were to become available in the future, the same methodology could be easily applied to all relevant variables in our metrics and opportunity scores.


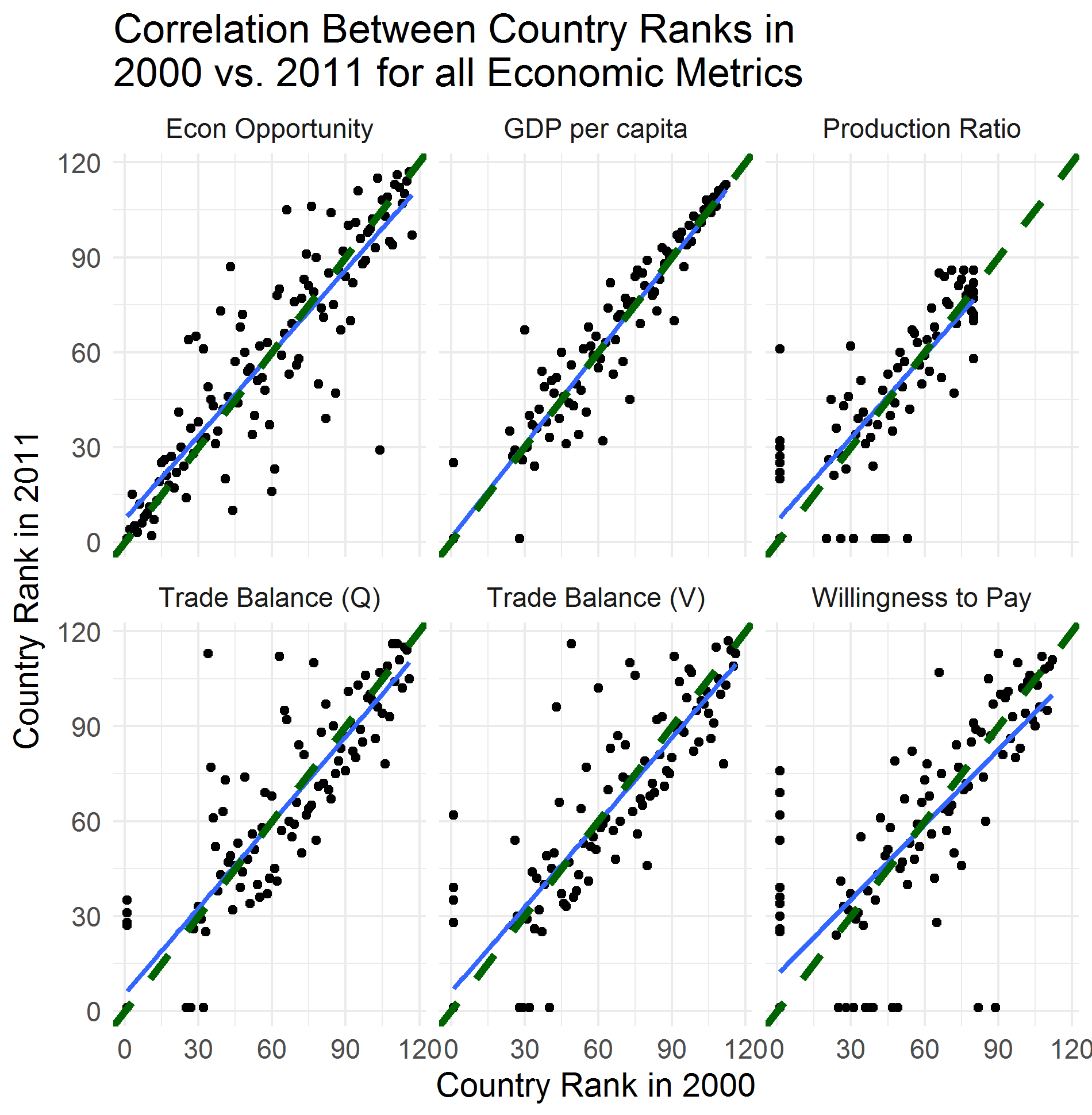


Figure A1: Relative change in the economic opportunity score for all countries from 2000 to 2011. The x axis represents a country’s year 2000 score, while the y axis shows it’s ranking in 2011. Each point denotes an individual country. The green dotted line is the 1:1 line (along which a country’s relative rank in 2000 is equal to its rank in 2011), while the blue line denotes the best linear fit across all observations. Each panel shows the analysis for a given economic metric.

Table A1: Kendall’s W test for rank concordance across economic opportunity metrics and scores, between pairs of data years. Kendall’s W statistic ranges from zero to one, with one indicating complete concordance and zero being complete disagreement. Significant P-values indicate concordance.

|  | **2000-2005** | | **2000-2011** | | **2005-2011** | |
| --- | --- | --- | --- | --- | --- | --- |
| **Item** | **Kendall's W** | **P-value** | **Kendall's W** | **P-value** | **Kendall's W** | **P-value** |
| GDP per capita | 0.994 | 2.88E-09 | 0.983 | 5.43E-09 | 0.991 | 3.52E-09 |
| Production Ratio | 0.942 | 7.10E-07 | 0.931 | 1.15E-06 | 0.965 | 2.56E-07 |
| Trade Balance (Q) | 0.948 | 2.24E-08 | 0.948 | 2.27E-08 | 0.954 | 1.56E-08 |
| Trade Balance (V) | 0.969 | 6.94E-09 | 0.944 | 2.78E-08 | 0.958 | 1.28E-08 |
| Willingness to Pay | 0.953 | 2.85E-08 | 0.906 | 3.55E-07 | 0.953 | 2.93E-08 |
| Economic opportunity | 0.945 | 2.34E-08 | 0.938 | 3.55E-08 | 0.925 | 7.18E-08 |

***Seafood reliance versus seafood preference***

A second limitation of our main analysis relates to the difficulty of differentiating between pure reliance on seafood and seafood preferences within a nation. This problem arises from the fact that our data source for the seafood reliance metrics (GeNUS) simply models revealed diets, and does not distinguish between necessity (availability) and choice. If reliance and preference are indeed intertwined, the interpretation of final mariculture opportunity scores becomes more nuanced.

To further examine this potential effect, we explored two dimensions of the countries in our sample: i) geographic characteristics, and ii) income level. The first refinement establishes if countries are islands or continental nations. One of the patterns observed in our main results is that island nations show a consistently higher overall mariculture potential. It is possible that this result could be mostly driven by seafood reliance (and/or seafood preference), and breaking down the analysis in this manner should allow us to parse out the influence of the seafood reliance versus nutritional and economic scores on the overall higher mariculture opportunity scores of island nations.

Figure A2 reports the results of imposing this first refinement on analysis. Note from the figure that island nations, regardless of their seafood reliance score, show consistently higher economic and nutritional opportunities. Continental countries on the other hand, show a higher degree of dispersion, with most of them having more economic than nutritional opportunity. These patterns highlight the observation that island nations, which naturally rely more on seafood, also present a consistent high nutritional and economic opportunity. It is expected then, that these nations would be able to capture most of the benefits of mariculture development, both in terms of nutrition and economic opportunity.


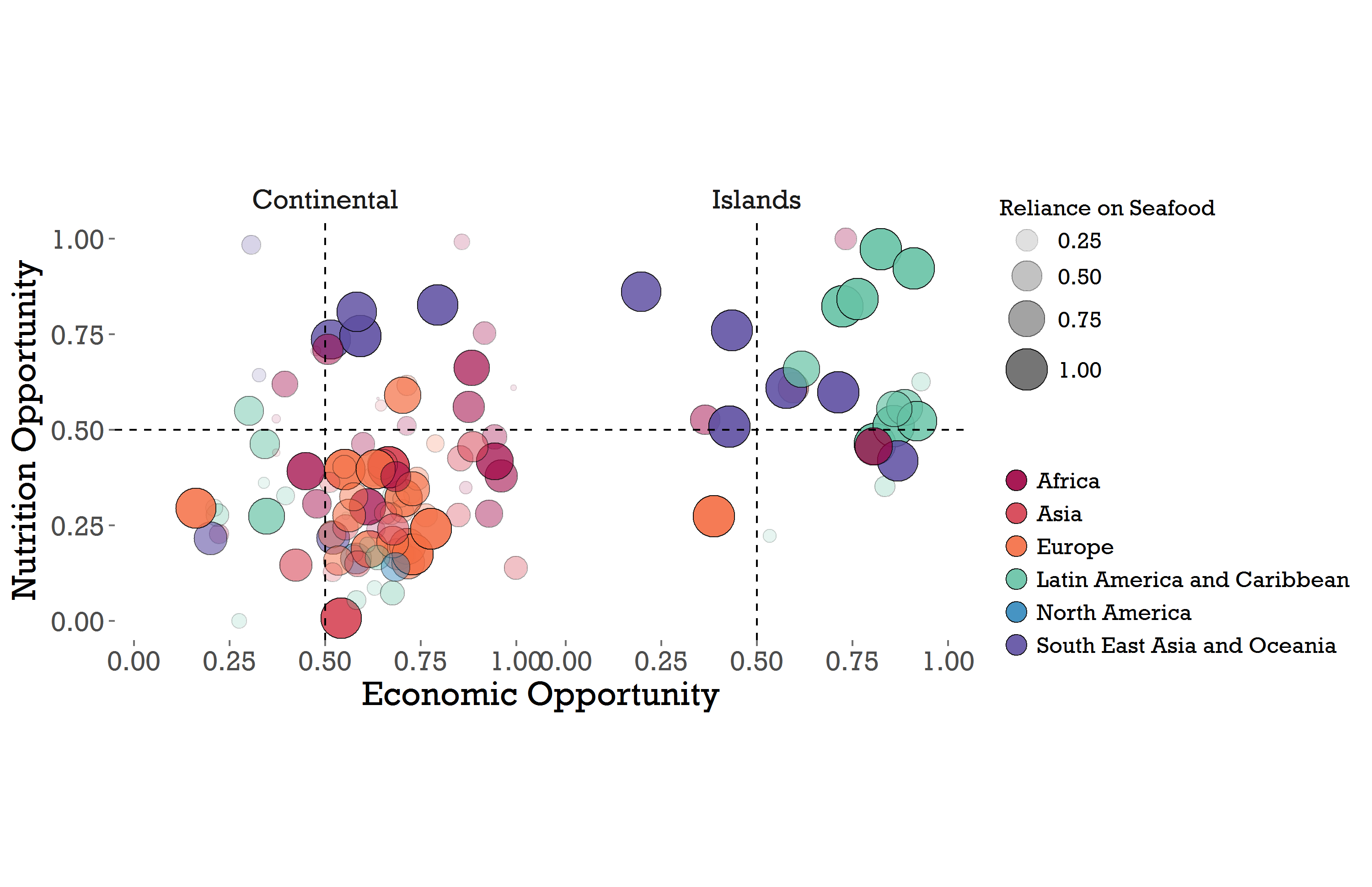


*Figure A2: Results of global analysis of nutritional opportunity, seafood reliance, and economic opportunity, for continental (left panel) and island (right panel) nations. Individual countries (n=117) scatter along economic and nutritional opportunity scores on the x and y axes, respectively, where each point indicates the performance of a given country. Scores are scaled from zero to one (see Methods, main text), such that countries in the upper right quadrant have both a high economic and nutritional opportunity for mariculture development. Size and opacity of country points scale with each country’s seafood reliance score, while color indicates a country’s geographic region.*

The second refinement explores how nations’ income levels affect the results for all of our metrics of interest. This problem is especially relevant for our reliance metric. It could be argued that rich countries do not necessarily rely on seafood, but rather choose their level of consumption, and these countries could bias overall results through their seafood preference rather than reliance. To shed some light on the general distribution of our scores, we split the analysis by income level as defined by the World Bank (a metric external to our other, included economic variables) to establish how our main observations and conclusions change under this new perspective.

Figure A3 shows the results of imposing this second refinement. Figure A3A shows how the overall score of mariculture opportunity depends on income level. The distribution of countries is fairly stable across groups, and the post-hoc Tukey test shows no statistically significant differences across income levels (Shaffer 1995). Figure A3B, on the other hand, shows the distribution of final nutritional opportunity score across income groups. As expected, low income countries show a statistically higher mean than the high-income group, while lower- middle and upper-middle countries are non-differentiable from each other and from either low or high income nations. This result is relatively intuitive: as countries increase their income levels, their population is able to cover their nutritional needs and thus have a low opportunity along that dimension. There is also no significant difference in economic opportunity score between income groups (Fig. A3C). Many countries have an economic opportunity to develop mariculture, regardless of current income.

Fig. A3D shows the exercise for our seafood reliance score. This analysis shows no statistically significant differences between means for all groups, except for the high income group. Seafood reliance seems to be skewed toward richer countries, indicating a potential influence of seafood preference (a choice to eat seafood) rather than pure dietary reliance, on our main results.


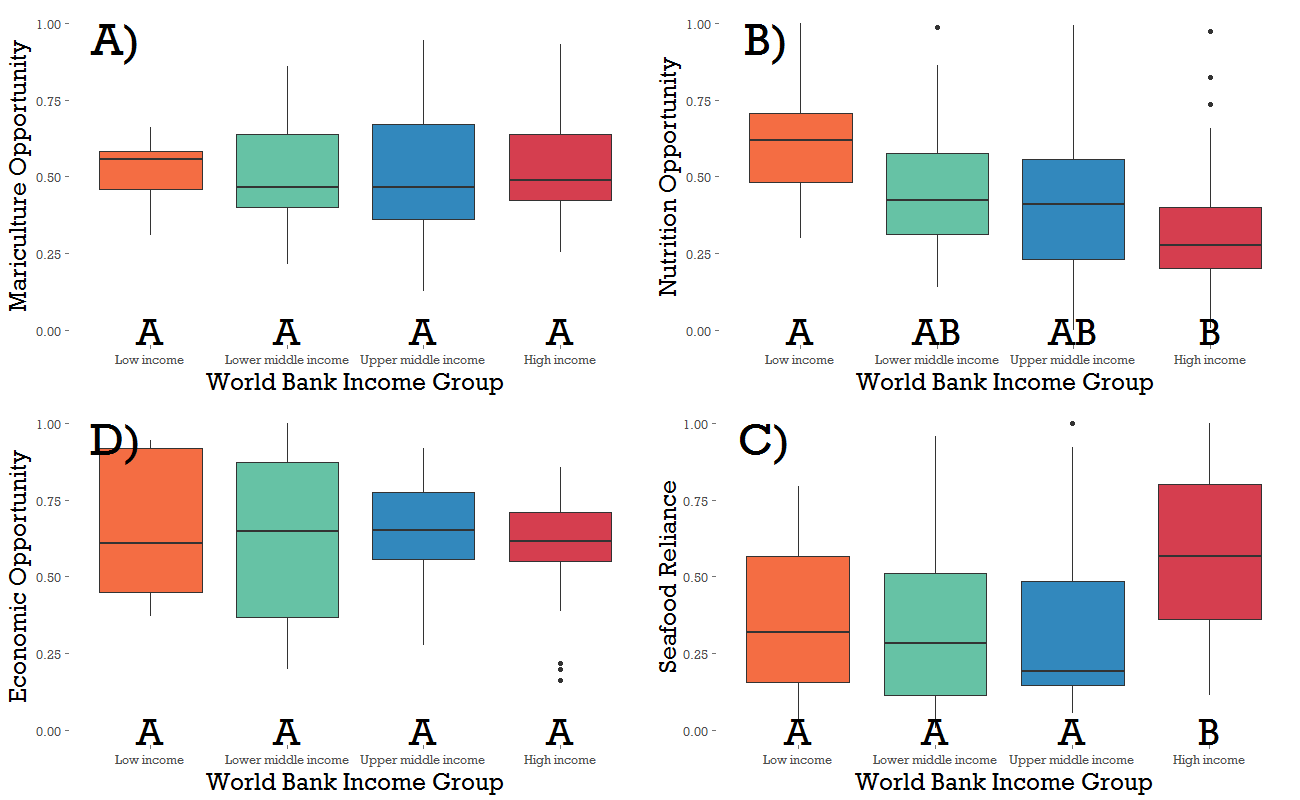


Figure A3: Distribution of opportunity scores as a function income level as defined by the World Bank. A) mariculture opportunity; B) nutrition opportunity; C) seafood reliance; and D) economic opportunity score distributions. Letters below each box are the ANOVA group differences after post-hoc Tukey HSD test.

This observation suggests that our reliance metric is likely capturing a combination of seafood availability, accessibility, and preference, effects which are not separable in our data, suggesting two additions to our interpretation of our main results. First, although an equal component in our overall mariculture opportunity score, reliance can also be viewed as a secondary filter in interpretation; that is, we endeavor to identify countries with high economic and nutritional opportunity, and within that set of nations existing presence of seafood in the diet will only bolster that opportunity. Secondly, the observed relationship in Figure A3D suggests a positive link between development (measured as increased per capita income) and seafood preference. So, for low income countries (which show a significantly higher relative nutritional opportunity), it is possible that increases in local production will result in higher seafood intake despite having low reliance as of 2011. This is further (although correlative) evidence that capturing nutritional and economic benefits could be positively reinforcing efforts.

***Sensitivity analyses***

To further evaluate the robustness of our results, we have also conducted a series of sensitivity analyses to determine the sensitivity of final mariculture opportunity scores to relative zero to one rescaling of individual metrics. Table A2 shows a sensitivity analysis for the normalizing decile (80^th^, 90^th^ or 100^th^ percentile used for normalization of metrics). Importantly, the rankings produced from these different normalization choices were significantly concordant, as mentioned in the main text. Finally, Table A3 shows the sensitivity of overall opportunity scores to the removal of individual metrics.

Table A2: Sensitivity of final mariculture opportunity scores to relative zero to one rescaling of individual metrics. Top 10 and bottom 10 countries shown for each choice of rescaling. Overall concordance between the rankings produced by the three scaling choices was high and significant (Kendall’s W 0.925; p<<0.05).

|  | **Normalization Threshold** | | |
| --- | --- | --- | --- |
| **Final Opportunity  Score Rank** | **Simple** | **90^th^ percentile** | **80^th^ percentile** |
| **1** | Maldives | Saint Lucia | Saint Lucia |
| **2** | Saint Kitts and Nevis | Saint Kitts and Nevis | Saint Kitts and Nevis |
| **3** | Saint Lucia | Grenada | Grenada |
| **4** | Grenada | Sri Lanka | Sri Lanka |
| **5** | Sri Lanka | Malaysia | Bahamas |
| **6** | Bahamas | French Polynesia | Barbados |
| **7** | Malaysia | Bahamas | Jamaica |
| **8** | French Polynesia | Barbados | Malaysia |
| **9** | Libya | Antigua and Barbuda | Thailand |
| **10** | New Caledonia | New Caledonia | French Polynesia |
| **108** | Honduras | Iran | Pakistan |
| **109** | Uruguay | Brazil | Cuba |
| **110** | Brazil | Cuba | Turkey |
| **111** | Costa Rica | Nicaragua | Mexico |
| **112** | Argentina | Mexico | Brazil |
| **113** | United States | Chile | Chile |
| **114** | Kuwait | Pakistan | Honduras |
| **115** | Turkey | Turkey | Nicaragua |
| **116** | Mexico | Mauritania | Mauritania |
| **117** | Mauritania | Argentina | Argentina |

Table A3: Results of sensitivity analysis investigating the impact of the exclusion of individual variables on opportunity scores. Numbers represent the mean change in economic opportunity, nutritional opportunity, and seafood reliance scores, as well as overall mariculture opportunity score across countries (columns) when the global analysis is re-run without each variable (rows). Standard deviations are in parentheses.

|  | **Mean Change** | |  | **Mean Change** | |  | **Mean Change** | |
| --- | --- | --- | --- | --- | --- | --- | --- | --- |
| **Variable removed** | **Nutrition opportunity** | **Mariculture opportunity** | **Variable removed** | **Seafood reliance** | **Mariculture opportunity** | **Variable removed** | **Economic opportunity** | **Mariculture opportunity** |
| Energy adequacy | 0.002 | 0.001 | Seafood Calories | -0.005 | -0.002 | Production ratio | -0.012 | -0.004 |
|  | (0.030) | (0.010) |  | (0.013) | (0.004) |  | (0.084) | (0.028) |
|  |  |  |  |  |  |  |  |  |
| Protein | 0.003 | 0.001 | Seafood protein | -0.003 | -0.001 | Trade balance (Q) | -0.013 | -0.004 |
|  | (0.041) | (0.014) |  | (0.018) | (0.006) |  | (0.065) | (0.022) |
|  |  |  |  |  |  |  |  |  |
| Fatty acids | -0.035 | -0.012 | Seafood PUFAs | 0.001 | 0.000 | Trade balance (V) | -0.012 | -0.004 |
|  | (0.061) | (0.020) |  | (0.028) | (0.009) |  | (0.066) | (0.022) |
|  |  |  |  |  |  |  |  |  |
| Vitamin A | -0.025 | -0.008 | Seafood vitamin A | 0.008 | 0.003 | Willingness to pay | 0.012 | 0.004 |
|  | (0.081) | (0.027) |  | (0.036) | (0.012) |  | (0.077) | (0.026) |
|  |  |  |  |  |  |  |  |  |
| Zinc | -0.005 | -0.002 | Seafood zinc | 0.001 | 0.000 | GDP per capita | 0.025 | 0.008 |
|  | (0.038) | (0.013) |  | (0.022) | (0.007) |  | (0.098) | (0.033) |
|  |  |  |  |  |  |  |  |  |
| Iron | -0.014 | -0.005 | Seafood iron | -0.002 | -0.001 |  |  |  |
|  | (0.065) | (0.022) |  | (0.027) | (0.009) |  |  |  |

***References***

Kendall, M. G. (1955). Rank correlation methods (2nd ed.). Oxford, England: Hafner Publishing Co.

Shaffer, J. P. (1995). "Multiple hypothesis testing." *Annual Review of Psychology* **46**: 561-584.
